# Risk of suicide following an alcohol-related emergency hospital admission: An electronic cohort study of 2.8 million people

**DOI:** 10.1371/journal.pone.0194772

**Published:** 2018-04-27

**Authors:** Bethan Bowden, Ann John, Laszlo Trefan, Jennifer Morgan, Daniel Farewell, David Fone

**Affiliations:** 1 Public Health Wales NHS Trust, Swansea, United Kingdom; 2 Swansea University Medical School, Institute of Life Sciences 2, Swansea, United Kingdom; 3 Division of Population Medicine, School of Medicine, Cardiff University, Cardiff, United Kingdom; Yokohama City University, JAPAN

## Abstract

**Objective:**

Alcohol misuse is a well-known risk factor for suicide however, the relationship between alcohol-related hospital admission and subsequent risk of death from suicide is unknown. We aimed to determine the risk of death from suicide following emergency admission to hospital with an alcohol-related cause.

**Methods:**

We established an electronic cohort study of all 2,803,457 residents of Wales, UK, aged from 10 to under 100 years on 1 January 2006 with six years’ follow-up. The outcome event was death from suicide defined as intentional self-harm (ICD-10 X60-84) or undetermined intent (Y10-34). The main exposure was an alcohol-related admission defined as a ‘wholly attributable’ ICD-10 alcohol code in the admission record. Admissions were coded for the presence or absence of co-existing psychiatric morbidity. The analysis was by Cox regression with adjustments for confounding variables within the dataset.

**Results:**

During the study follow-up period, there were 15,546,355 person years at risk with 28,425 alcohol-related emergency admissions and 1562 suicides. 125 suicides followed an admission (144.6 per 100,000 person years), of which 11 (9%) occurred within 4 weeks of discharge. The overall adjusted hazard ratio (HR) for suicide following admission was 26.8 (95% confidence interval (CI) 18.8 to 38.3), in men HR 9.83 (95% CI 7.91 to 12.2) and women HR 28.5 (95% CI 19.9 to 41.0). The risk of suicide remained substantial in subjects without known co-existing psychiatric morbidity: HR men 8.11 (95% CI 6.30 to 10.4) and women HR 24.0 (95% CI 15.5 to 37.3). The analysis was limited by the absence in datasets of potentially important confounding variables and the lack of information on alcohol-related harm and psychiatric morbidity in subjects not admitted to hospital.

**Conclusion:**

Emergency alcohol-related hospital admission is associated with an increased risk of suicide. Identifying individuals in hospital provides an opportunity for psychosocial assessment and suicide prevention of a targeted at-risk group before their discharge to the community.

## Introduction

In the UK, suicide is the leading cause of death for people aged 20–35 years and for men aged 35–49 years [1]. Men remain three times more likely than women to take their own lives [2] but female suicide rates are rising, currently at their highest in a decade [3].^-^Alcohol use can increase in response to deterioration in psychological wellbeing [4], and misuse is a predisposing factor for suicide [5] with a lifetime prevalence of suicide of 7% in people with alcohol dependence in comparison with 0.7% in the general population [6]. Consumption of alcohol immediately prior to suicide is common [7,8], with an estimated 37% of deaths from suicide having positive blood alcohol concentrations on toxicology screening indicating acute alcohol consumption before death [9]. Given this relationship between alcohol consumption and suicide, it is possible that an alcohol-related emergency hospital admission could be a useful marker for a higher risk of suicide and a key moment for intervention to prevent future suicidal behaviour. We aimed to investigate the association between suicide and the full spectrum of alcohol-related acute hospital admissions. We were able to achieve this aim by quantifying the risk of suicide following all alcohol-related acute hospital admissions in a total population cohort of 2.8 million people in Wales, UK.

## Methods

### Study population

The study design was an electronic record-linked retrospective population cohort. Participants were from the ‘Change in alcohol outlet density and alcohol-related harm to population health’ (CHALICE) study [10]. The cohort in this analysis comprised all persons aged from 10 years to under 100 years who were resident in Wales, UK, on 1st January 2006 (n = 2,803,457) with six-year follow-up to 31st December 2011. The CHALICE cohort is held in the Secure Anonymised Information Linkage (SAIL) databank at Swansea University, UK [11,12], one of four Centres in the UK Farr Institute of Health Informatics Research [13]. The study was approved by the SAIL Databank Information Governance Review Panel, who confirmed that the study met the strict information governance arrangements set out in the multiple data access agreements, ensured anonymity and did not require referral to the National Research Ethics Service as the data were de-identified.

### Data sources

The Welsh Demographic Service (WDS) is the core demographic dataset on the total population of Wales registered with a general medical practice. It includes information on age, sex and the UK Office for National Statistics (ONS) Census lower layer super output area (LSOA) place of residence. With a mean population of 1500 people, the LSOA is the smallest geographical area used for reporting UK Census statistics that can be used in the SAIL databank. There were 1896 LSOAs in Wales defined in the 2001 UK Census in use during the study period. Each subject was assigned by LSOA to a quintile of the Welsh Index of Multiple Deprivation (WIMD) [14] and to the ONS 2004 residential settlement Rural-Urban categorisation [15]. This demography file was linked using a unique person-level anonymised identifier to the Patient Episode Database for Wales (PEDW), which includes administrative and clinical information on all inpatient and day-case admissions in Welsh hospitals and those of Welsh residents admitted to hospitals in England. The file was also linked to the ONS Annual District Death Extract for information on the date and the underlying cause of death coded using the International Classification of Disease, revision 10, (ICD-10) [16].

### Definition of outcome variable

The primary outcome was death from suicide. This was defined using ICD-10 codes for death by intentional self-harm (X60-84) and deaths from undetermined intent (Y10-34) in accordance with ONS reporting practice to prevent under-estimation through misclassification [17]. The definition of suicide in subjects aged 10 to 14 years included intentional self-harm only [3]

### Definition of exposure variables

The primary exposure was an alcohol-related emergency admission. An alcohol-related admission was defined as the first emergency admission in the study period with an alcohol-related ICD-10 code populating one of the first three coding positions in the admission record [10]. We defined an alcohol-related ICD-10 code as being ‘wholly attributable’ to alcohol consumption [10] and categorised them into clinical groups of interest based on the ICD-10 sections of (1) Mental and behavioural disorders related to alcohol (2) Toxic effects of alcohol & poisoning by alcohol and (3) Diseases of the digestive system, encounters with alcohol services or due to alcohol use, and ‘other’ (Table 1). If there was no diagnostic code in the first three positions, for example where symptoms or contact with services were coded as the reason for admission, then a diagnostic alcohol code when present in the 4th position was included in the definition.

**Table 1:** ICD-10 defined conditions defined as wholly attributable to alcohol.

Study category	ICD-10 code
Group 1: Mental and behavioural disorders due to use of alcohol	
Acute intoxication	F10.0
Alcohol use disorder: Harmful use and Dependence	F10.1, F10.2
Withdrawal state; Withdrawal state with delirium; Psychotic disorder; Amnesic syndrome; Residual and late-onset psychotic disorder; Other mental and behavioural disorders; Unspecified mental and behavioural disorder	F10.3, F10.4, F10.5, F10.6, F10.7, F10.8, F10.9
**Group 2: Toxic effects of alcohol & poisoning by alcohol**	
Toxic effect: Ethanol	T51.0
Accidental or intentional poisoning by and exposure to alcohol	X45, X65
Evidence of alcohol involvement determined by blood alcohol level	Y15, Y90, Y91
Group **3: Diseases of the digestive system, encounters with alcohol services or due to alcohol use, and ‘other’**	
Diseases of the digestive system–alcoholic gastritis, alcoholic liver disease and pancreatitis;;	K29.2, K70.0—K70.4, K70.9, K85, K86
Alcohol-induced pseudo-Cushing's syndrome, Wernicke's encephalopathy	E24.4, E51.1,
Degeneration of nervous system due to alcohol, Special epileptic syndromes–paired with other alcohol code, Alcoholic myopathy;	G31.2, G40.5, G62.1, G72.1,
Alcoholic cardiomyopathy; Maternal care for (suspected) damage to foetus from alcohol	I42.6, O35.4,
Finding of alcohol in blood; Alcohol rehabilitation, Alcohol abuse counselling and surveillance, Alcohol use	R78.0, Z50.2, Z71.4, Z72.1

Potential confounders included in the analyses were sex (male or female), age group on admission to hospital (10–14, 15–24, 25–34, 35–44, 45–54, 55–64, 65–74, 75–84, or 85–99 years), quintile of socioeconomic deprivation (lowest, low, middle, high or highest deprivation) and three residential settlement types (1) urban, (2) rural—town and fringe, or (3) village, hamlet and isolated dwellings. These variables were fully coded in the cohort dataset.

To assess the influence of known co-existing psychiatric morbidity in those admitted with an alcohol-related admission, we extracted codes for ‘Mental and behavioural disorders’ (ICD-10 Chapter F00 –F09, F11- F99, excluding alcohol-related causes, F10) where present in any coding position in the admission record. We then derived a three-part categorical variable of (1) no admission, (2) admitted without co-existing psychiatric morbidity, and (3) admitted with co-existing psychiatric morbidity. We were not able to extract mental health codes for the comparator group not admitted with an alcohol-related admission as information on mental health co-morbidities, including alcohol-use disorder, was not available within the dataset.

### Statistical analysis

Baseline characteristics were described using descriptive statistics and suicide incidence rates with exact confidence intervals from the Poisson distribution were calculated for males and females using the total person years at risk (PYAR) as the denominator for each age group, socioeconomic deprivation group and residential settlement type, and for each defined category of alcohol-related admission. Survival time in days, from alcohol admission to death from suicide, were calculated and cumulative percentage of suicides in the weeks post discharge were determined.

Hazard ratios (HR) with 95% confidence intervals (CI) for the risk of suicide were estimated using Cox regression models for the alcohol-related admission variables, stratifying by sex and adjusting for age group, socioeconomic deprivation and settlement type. The comparator group were all Welsh residents who were not admitted with an emergency alcohol-related admission. Models were censored for migration out of the study area and death from another cause. As the hospital admission could occur at any time during the six-year follow-up we modelled this as a time-dependent variable. We tested the proportional hazards assumption using both a formal significance test based on Schoenfeld residuals [18], along with plots of both the Schoenfeld residuals and cumulative hazards. All analysis was performed in IBM SPSS statistics v20 [19] and R software [20] remotely through the SAIL Gateway to access the study cohort in the SAIL databank.

## Results

**Fig 1:**
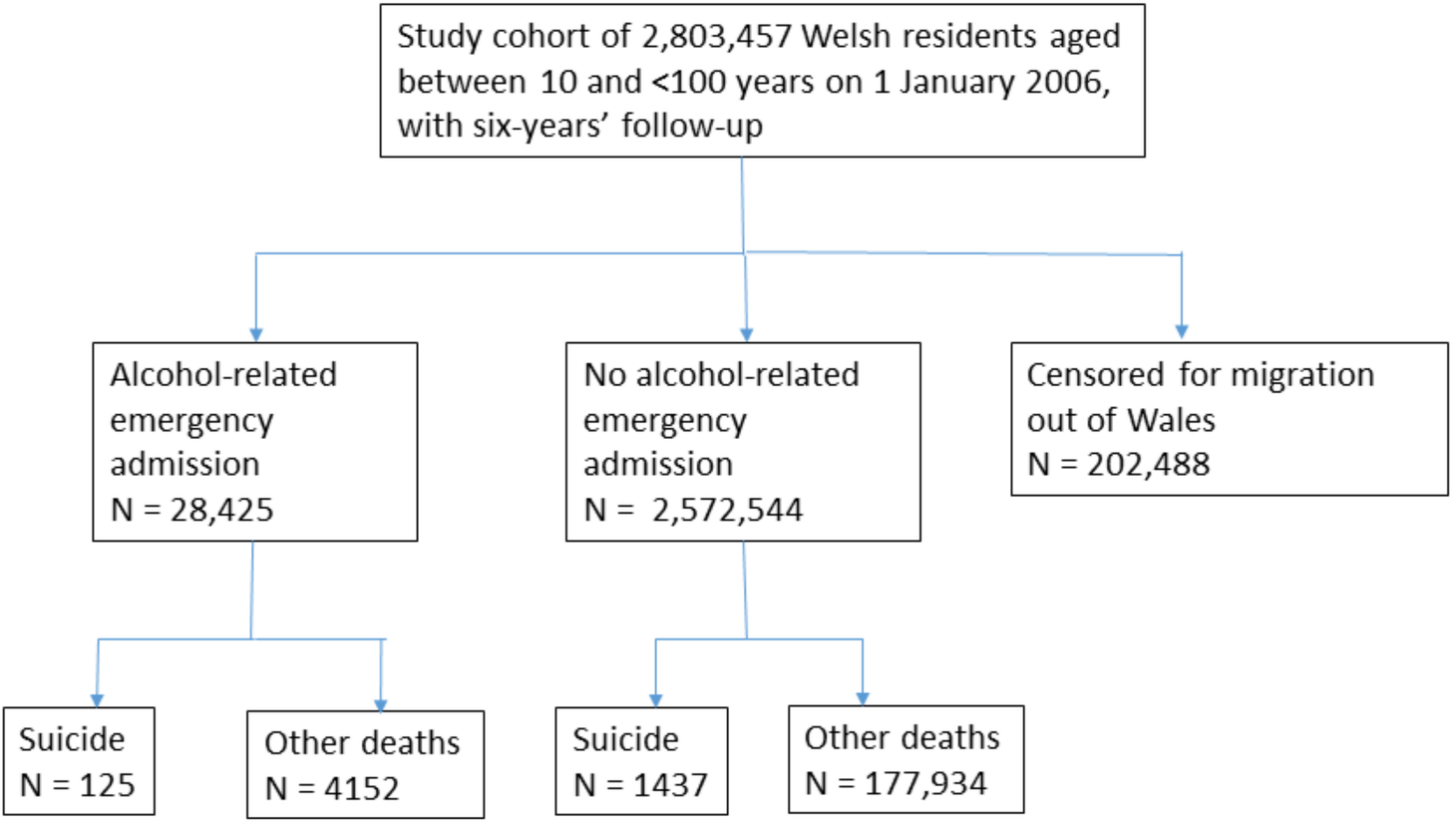
Study flow chart.

During the six-year study period, the 2,803,457 Welsh residents aged from 10 to under 100 years contributed a total of 15,546,355 person years at risk (mean = 5.55 years). The study flow chart is shown in Fig 1. There were a total of 28,425 alcohol-related admissions with an incidence rate of 183 per 100,000 PYAR (95% CI: 181, 185). The incidence rate was higher in all age groups for males compared to females apart from subjects aged under 16 years. Admission rates were highest in middle-age (35–54 years), in residents of urban or town environments and of the most disadvantaged communities in Wales (S1 Table).

There were 1562 suicides during the study period with an incidence rate of 10.0 per 100,000 PYAR (95% CI: 9.51, 10.5) (S1 Table). The incidence of suicide for men of 15.8 per 100,000 PYAR was substantially higher than for women at 4.40 per 100,000 PYAR (crude incidence ratio 3.59, 95% CI: 3.19, 4.05). The highest incidence rates of suicide were found in the middle age groups of 35–54 years, in the most deprived areas and in urban settlements (S1 Table).

The total number of deaths in the cohort was 183,648. Of these, 2206 (1.2%) did not have a cause of death recorded. Death from suicide occurred in 125 subjects following an alcohol-related admission at 144.6 per 100,000 PYAR, compared to 1437 suicides in subjects not admitted at 9.29 per 100,000 PYAR, (Table 2), risk ratio 15.6 (95% CI: 13.0, 18.7). The highest incidence of suicide (qualified by five or more deaths) was seen in the alcohol use disorder and ‘toxic effects and poisoning by alcohol’ admission groups (Table 2).

**Table 2:** Frequency and incidence rates of death from suicide by alcohol admission codes.

	Males		Females		Total	
Alcohol admission diagnostic category	Frequency (n)	Incidence rate (per 100,000 PYAR)	Frequency (n)	Incidence rate (per 100,000 PYAR)	Frequency (n)	Incidence rate (per 100,000 PYAR)
No admission	1125	14.7	312	3.99	1437	9.29
All alcohol-related codes	91	163.3	34	110.8	125	144.6
Alcohol code diagnostic category						
F10.0	15	100.7	6	71.9	21	90.4
F10.1–10.2	35	224.8	10	142.8	45	199.4
F10.3—F10.9	*	104.2	*	209.9	9	134.3
Toxic effects of alcohol (T-codes) & poisoning by alcohol (XY-codes)	20	312.4	10	134.5	30	216.8
Diseases of digestive system (K-codes), encounters with alcohol services or due to alcohol use (Z-codes), & other codes	*	113.8	*	66.6	20	99.7
Alcohol-related without mental health code	65	133.1	22	91.1	87	119.2
Alcohol-related with mental health code	26	376.8	12	183.5	38	282.7

* = withheld due to small numbers <5

Fewer than five of the 125 deaths occurred in hospital, all coded as Y109 (Poisoning, undetermined intent). Of the deaths occurring post-discharge the time from alcohol-related admission to death from suicide ranged from 2 days to 2096 days. Five deaths (4.1%) occurred within one week of discharge with a cumulative total of eleven deaths (8.9%) within 4 weeks of discharge.

Of the 28,425 alcohol-related admissions, 4478 (15.8%) subjects had at least one ‘Mental and behavioural disorders’ ICD-10 code (excluding F10) in their admission record. A total of 38 deaths from suicide were ascertained in this group, of which 26 admissions were coded with F30-F39 ‘Mood [affective] disorders’ and seven with F40-F48 ‘Neurotic, stress-related and somatoform disorders.’ All other F-codes combined accounted for the remaining five deaths. The incidence risk ratio for death from suicide in subjects with coded co-existing psychiatric morbidity compared to those admitted without was 2.37 (95% CI: 1.62, 3.47). (Table 2).

In a preliminary Cox model for males and females combined we found an alcohol-related admission from any cause was associated with a 27-fold increased risk of death from suicide (HR 26.8, 95% CI 18.8 to 38.3) (S2 Table). The risk of death from suicide increased significantly for both males and females with increasing socioeconomic deprivation, but no significant risk was associated with residence in a rural town or village area compared to urban (S2 Table). In this model we found a significant interaction between sex and alcohol-related admission, and so we modelled the risk of suicide separately for men and women.

In the stratified models, the risk was 3-times higher in women (HR 28.5, 95% CI 19.9 to 41.0) than men (HR 9.83, 95% CI 7.91 to 12.2) (Table 3). Significantly higher risks were found in males aged 25 to 54 and females aged 35 to 64 years and 75 to 84 years in comparison to the reference group aged 16 to 24 years (S2 Table). Interactions between admission and age group, deprivation, or settlement type, were not statistically significant.

**Table 3:** Adjusted risk of death from suicide for categories of alcohol-related admission, stratified by sex.

	Males				Females			
Alcohol admission diagnostic category	Adjusted HR^a^	95.0% CI for HR		p-value	Adjusted HR^a^	95.0% CI for HR		p-value
		Lower	Upper			Lowerr	Upper	
No admission	Reference				Reference			
Any alcohol-related admission	9.83	7.91	12.2	<0.001	28.5	19.9	41.0	<0.001
F10.0	5.29	3.4	8.25	<0.001	21.9	10.8	44.4	<0.001
F10.1—F10.2	9.81	7.44	12.9	<0.001	23.8	14.2	40.1	<0.001
F10.3–10.9	8.88	4.6	17.1	<0.001	37.4	13.9	100.6	<0.001
T/XY	17.9	13.4	23.8	<0.001	29.8	19.3	46.1	<0.001
K/Z/other	3.74	2.62	5.36	<0.001	6.94	3.09	15.6	<0.001
Alcohol-related without mental health code	8.11	6.3	10.4	<0.001	24.0	15.5	37.3	<0.001
Alcohol-related with mental health code	21.1	14.2	31.1	<0.001	43.6	24.4	78.1	<0.001

^a^ = HR adjusted for age, sex, residential settlement and WIMD

**Fig 2:**
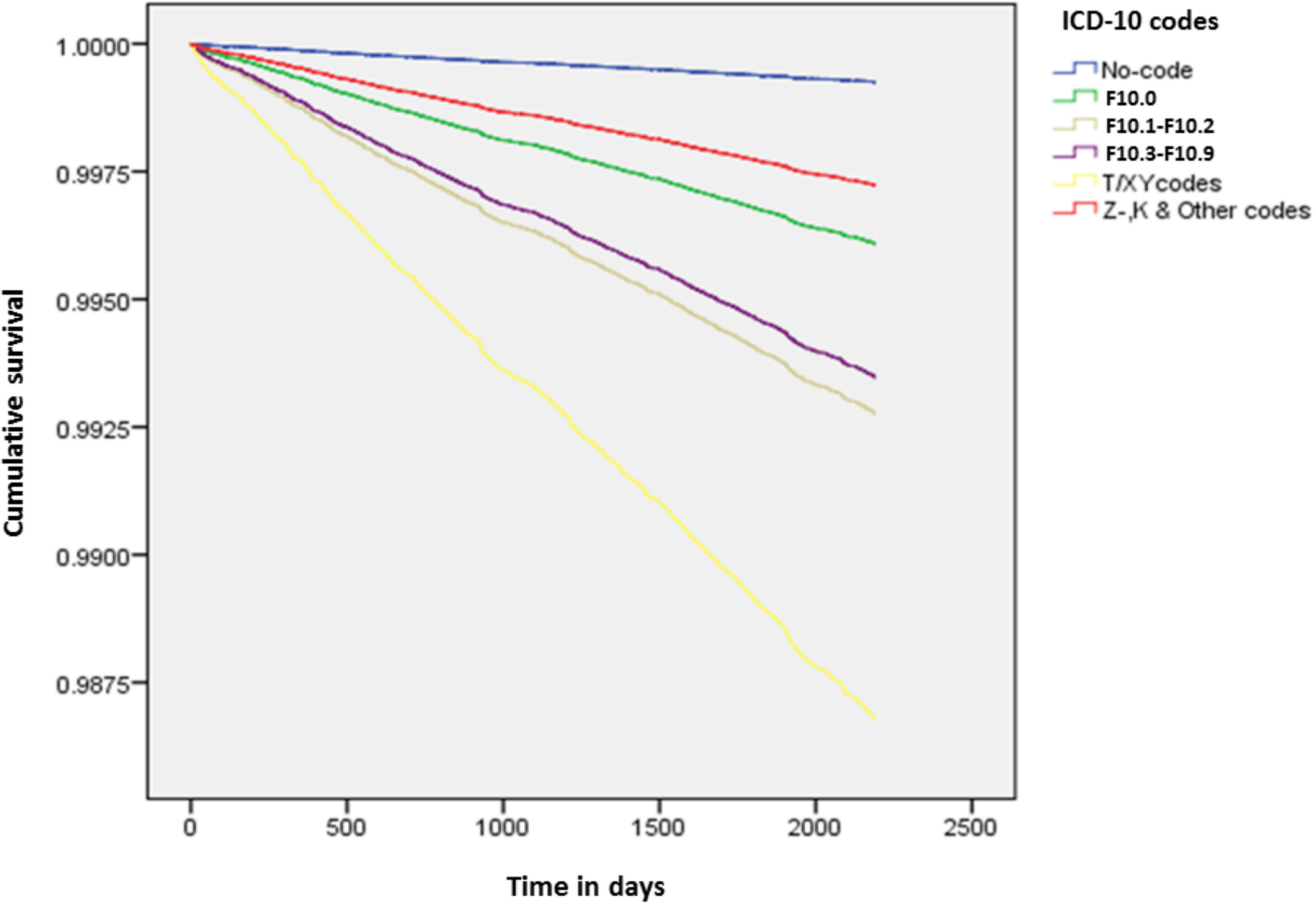
Cumulative survival plot for diagnostic categories of alcohol-related admissions: Males.

**Fig 3:**
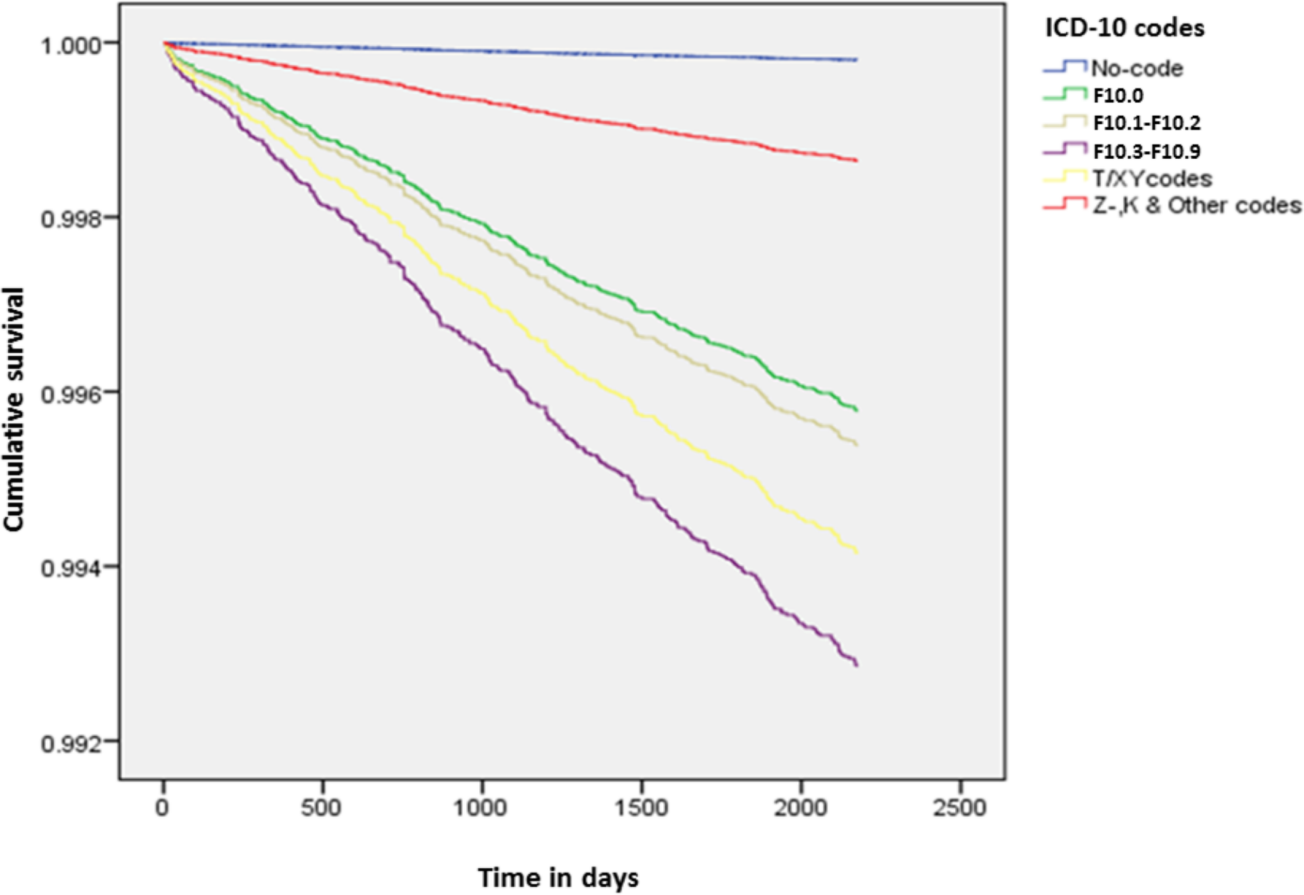
Cumulative survival plot for diagnostic categories of alcohol-related admissions: Females.

The adjusted risk of suicide varied with the clinical type of alcohol-related admission, and all risks were substantially higher in women (Table 3), (Figs 2 and 3). The highest risks (for five or more deaths) were associated with admission for ‘toxic effects of alcohol or poisoning by alcohol’ where the risk of suicide was 18-times higher than for those not admitted in men and 30-times higher in women, followed by alcohol use disorder at nearly 10-fold and 24-fold, respectively. Admission with acute alcoholic intoxication was associated with a 5-fold increased risk of suicide in men and 22-fold increase in women. Among men, the risk associated with alcohol use disorder increased to 10-fold, whereas in women the risk was similar to the risk associated with acute intoxication.

We found a substantially higher 43-fold risk of suicide in women and 21-fold in men with an alcohol-related admission and a pre-existing psychiatric diagnosis (Table 3). Patients admitted with no known psychiatric morbidity were still found to have a substantial risk compared to subjects not admitted, at 8-fold and 24-fold in men and women, respectively.

Model checking of the Schoenfeld residuals found that none of the models violated the proportional hazards assumption.

## Discussion

We found that individuals admitted as an emergency to hospital with an alcohol-related cause were a high-risk group for subsequent suicide and this risk was nearly 3-times higher overall in women than men. The strength of the association varied with the alcohol-related diagnosis on admission. We found the highest risk in the ‘toxic effects of alcohol or poisoning through alcohol’ group with a 30 and 18-fold increased risk in women and men, respectively. In the mental and behavioural disorders categories, the highest risk of suicide was markedly higher in women with alcohol use disorder at 24-fold, compared to men at 10-fold. However, patients admitted with acute intoxication only still had a markedly elevated risk at 22-fold in women and 5-fold in men, compared to subjects not admitted.

Patients with a co-existing psychiatric diagnosis and an alcohol-related admission were at highest risk of suicide. For women, the risk of suicide was 43-times higher than subjects not admitted. For men the risk was 21-times higher. The risk observed in patients admitted with no coded psychiatric illness was still substantial, at 24-times the risk of subjects not admitted in women and 8-times higher in men.

### Strengths and limitations

This was a large total population study of nearly 3 million participants followed for six years and the first UK study of which we are aware to investigate the risk of suicide following an alcohol-related emergency admission. The use of electronic record-linkage in this study resulted in complete information on age, sex, socioeconomic deprivation, residential settlement type and date of death or migration. Wales can be considered comparable with other countries within the United Kingdom and Northern Europe with similar levels of per-capita alcohol consumption [21], enabling generalisability of results to these populations.

Investigation of relatively uncommon outcomes such as suicide can be limited by small numbers. However, the use of a total population electronic cohort study design with over 15 million person-years at risk ensured adequate numbers of the primary outcome of suicide (n = 1562) to give reasonably precise estimates, although it was not possible to completely stratify by all the categories of alcohol and psychiatric codes by sex.

Although we achieved near complete ascertainment (98.8%) of the coded underlying cause of death to give high quality on the suicide primary outcome, a limitation is from a potentially small under-ascertainment of suicide in the un-coded deaths. Suspicious deaths require a coroner’s verdict on the cause of death and this may cause a delay between the date that death occurred and the registration of the death. In Wales, there was a median delay of 148 days in 2011 [3]. It is possible that the small proportion of missing cause of death records (1.2%) in the study includes those awaiting a coroner’s verdict of suicide or indeterminate intent. These registration delays are an inevitable limitation of study design with censoring at a fixed end point. A further under-enumeration of deaths from suicide may have occurred as ONS registry data may not record narrative verdicts. These are a factual reporting of the case by the coroner as suicide if they do not indicate the intent and mechanism of death [3]. Also excluded may be accidental deaths that on review by suicide researchers would be considered death from suicide [22].

The use of a narrow definition of alcohol-related admission was designed to avoid including admissions where alcohol was not the primary reason for admission. However, this involves a trade-off of specificity for sensitivity that may underestimate the overall burden of alcohol-related harm. Ethnicity [23], religion [23], marital status [23], chronic pain [24], physical illness [25], unemployment [26]^,^ and concomitant substance use [27] are known to be associated with suicide, but these variables could not be accounted for in this study as they are not recorded in the study datasets. Differences between countries in the distributions of these variables could limit the generalisability of our findings.

Mental disorders are a well-established risk factor for suicide [28] and we found, as expected, the highest risks of suicide in subjects with co-existing psychiatric co-morbidities. Although we were able to stratify the hospital admission by the presence or absence of a co-existing psychiatric diagnosis to estimate the risk of death from suicide separately for these groups, we had no information on psychiatric morbidity in subjects not admitted. This is likely to bias the study risk estimates towards the null, because the not admitted comparison group will include subjects with this higher risk. Further research should explore the potential of linking secondary care data with alcohol-related clinical coding held in primary care datasets [29]. This will enable a greater understanding of alcohol-related problems in the community and provide a more comprehensive source of information regarding psychiatric co-morbidities for future analyses. Our study only investigated the impact of an alcohol-related emergency hospital admission on future suicide deaths. We did not explore the potential for hospitalization from any other cause to be a risk factor for future suicide. Hospital admission following an acute mental health emergency or a drug-related emergency could be considered likely to also increase an individual’s risk of future suicide. Though an interesting focus for future research this data was not available within our datasets and could not be incorporated into our study. However, this should not reduce the magnitude of risk that we have identified in relation to an emergency alcohol-related admission. These potential confounding variables are not accounted for within our analysis but as these “higher risk” individuals remain within the general population comparator group, this would reduce our study risk estimates with the resultant direction of the bias towards the null.

### Comparison with published research

Two studies have investigated hospital admission or accident and emergency department attendance for alcohol-related causes and suicide. Both only investigated alcohol use disorder (AUD) in contrast to our total population study that included all alcohol-related diagnoses. One study, set in Denmark, followed 23,000 subjects in the Copenhagen City Heart Study for up to 26 years [30].Subjects with AUD, defined as ICD-10 F10.1 or F10.2, were identified from hospital admission or out-patient clinic registers. Suicide was defined as death from intentional self-harm only, and not including undetermined deaths as is conventional in the UK. After adjusting for sociodemographic factors the HR was smaller than in our study at 5.9 (95% CI: 3.8, 9.3). In a model adjusting for all psychiatric disorders the HR was 3.2 (95% CI: 2.0, 5.3). In an analysis stratified by psychiatric disorders, the risks were again smaller than our study, with the risk twice as high in subjects without a psychiatric disorder than in subjects with a disorder. This is in contrast to our findings of a higher risk of suicide in subjects with a psychiatric disorder, but consistent with an important elevated risk of suicide in patients with AUD and without known psychiatric illness.

A second study of AUD in Iceland identified 1200 patients with AUD among over 100,000 emergency department attendances with seven years’ follow-up [31]. AUD was associated with a higher risk of suicide, but with only 15 deaths from suicide the estimates were imprecise. An incidence risk was not presented in the paper but can be calculated at 263/100 000 PYAR (95% CI: 147, 434). This was higher than our study and may reflect different patterns of drinking between Iceland and Wales. The HRs, including adjustment for mental and behavioural disorders at discharge, for X60-X84 (2.78, 1.11 to 6.95) and Y10-Y34 (11.01, 4.60 to 26.3) were smaller than in our study for subjects without known psychiatric comorbidity.

### Clinical relevance

The substantial risk of suicide for all emergency patients admitted with an alcohol-related condition has implications for their clinical management. Alcohol misuse, from acute intoxication to alcohol dependence, is a well-known risk factor for suicide. We have found that patients admitted as an emergency with alcohol-related conditions have a substantially increased risk of subsequent suicide. This identifies a specific patient group within the general hospital setting for suicide prevention interventions. Men have a 3-fold higher risk of suicide than women in the general population, but women admitted with an alcohol-related condition have a 3-fold higher risk of death from suicide than men following admission and such women should be considered highly vulnerable. The patients at highest risk of suicide in our study were those admitted with an alcohol-related condition and psychiatric co-morbidity, patients likely to be already known to mental health services. However, we still found a substantial risk in patients admitted with no known psychiatric disorder. This group of patients are at risk of discharge without appropriate consideration of their mental wellbeing and the underlying psychological distress that led to the alcohol misuse [4]

Patients admitted to general hospitals with an alcohol-related disorder are typically managed by emergency and general physicians. Immediate management focuses on medical complications of alcohol misuse [32] with the assessment of the underlying mental state deferred until a patient reaches a sustained period of abstinence of 3 to 4 weeks [33]. We found that eleven of the 125 suicides that followed an alcohol-related admission occurred within four weeks of discharge. This indicates a missed opportunity to address the underlying psychological component associated with alcohol misuse. Repeated exposure to self-destructive events from excessive alcohol consumption has been shown to reduce the frequency of referral to specialised services by clinicians [32], but hospital admission provides an opportunity to engage with this high risk group.

Additionally, as death from suicide in the alcohol-admission group occurred throughout the 6 year follow up period, the identification of an emergency alcohol-related admission in an individual’s medical history can additionally be considered a marker for high-risk for suicide when assessing an individual in a community setting.

Our results suggests a need for all patients presenting with an alcohol-related emergency admission to undergo a comprehensive assessment of psychological distress and suicidal ideation, as conducted after an emergency admission for self harm, followed by sign-posting, support, treatment interventions and referral to mental health services, as appropriate [34]. This intervention should be evaluated to determine if the opportunity to improve access to mental health services before discharge in the high risk population identified in this study, reduces their future risk of suicide.

## Supporting information

S1 Table Frequency and incidence rates of alcohol-related admissions and suicide by sociodemographic variables. (DOCX)

S2 Table Adjusted risks of death from suicide, stratified by sex. (DOCX)

S1 File STROBE checklist cohort. (DOC)

## Abbreviations

AUD: alcohol use disorder
CHALICE: change in alcohol outlet density and alcohol-related harm to population health
CI: confidence interval
HR: hazard ratio
ICD-10: International Classification of Disease, revision 10
ONS: Office for National Statistics
PEDW: Patient Episode Database for Wales
SAIL: Secure Anonymised Information Linkage
WDS: Welsh Demographic Service
WIMD: Welsh Index of Multiple Deprivation
