# Supplementary material for: Risk of suicide following an alcohol-related emergency hospital admission: An electronic cohort study of 2.8 million people
Source: PLoS One. 2018 Apr 27;13(4):e0194772. doi: 10.1371/journal.pone.0194772 (PMC5922531; doi:10.1371/journal.pone.0194772)
Supplement: S1 Table — (DOCX) [file pone.0194772.s001.docx]

**S1 Table: Frequency and incidence rates of alcohol-related admissions and suicide by sociodemographic variables**

|  | **Alcohol-related admissions** | | | | | | | | **Suicide** | | | | | |
| --- | --- | --- | --- | --- | --- | --- | --- | --- | --- | --- | --- | --- | --- | --- |
|  | **Male** | | **Female** | | | **Total** | | | **Male** | | **Female** | | **Total** | |
| **Variable** | **Frequency (n)** | **Incidence rate (per 100,000 PYAR)** | **Frequency (n)** | **Incidence rate (per 100,000 PYAR)** | | **Frequency (n)** | | **Incidence rate (per 100,000 PYAR)** | **Frequency (n)** | **Incidence rate (per 100,000 PYAR)** | **Frequency (n)** | **Incidence rate (per 100,000 PYAR)** | **Frequency (n)** | **Incidence rate (per 100,000 PYAR)** |
| **Age group** | | | | | | | | |  | |  | |  | |
| **10-14** | 980 | 168.8 | 1150 | | 207.3 | 2130 | 187.6 | | 14 | 2.41 | 5 | 0.90 | 19 | 1.67 |
| **15-24** | 2961 | 248.9 | 1584 | | 138.9 | 4545 | 195.0 | | 156 | 13.1 | 36 | 3.16 | 192 | 8.24 |
| **25-34** | 2852 | 253.7 | 1350 | | 126.0 | 4202 | 191.4 | | 217 | 19.3 | 45 | 4.20 | 262 | 11.9 |
| **35-44** | 3837 | 285.8 | 2118 | | 162.3 | 5955 | 225.0 | | 323 | 24.1 | 62 | 4.75 | 385 | 14.5 |
| **45-54** | 3361 | 285.5 | 1718 | | 148.6 | 5079 | 217.6 | | 209 | 17.8 | 72 | 6.23 | 281 | 12.0 |
| **55-64** | 2766 | 248.4 | 1103 | | 98.3 | 3869 | 173.1 | | 146 | 13.1 | 54 | 4.81 | 200 | 8.95 |
| **65-74** | 1248 | 171.8 | 527 | | 66.0 | 1775 | 116.4 | | 76 | 10.5 | 35 | 4.38 | 111 | 7.28 |
| **75-84** | 468 | 127.5 | 283 | | 52.3 | 751 | 82.7 | | 63 | 17.2 | 31 | 5.73 | 94 | 10.4 |
| **Over 85** | 72 | 110.6 | 47 | | 27.5 | 119 | 50.5 | | 12 | 18.4 | 6 | 3.51 | 18 | 7.63 |
| **Residential settlement** | | | | | | | | |  |  |  |  |  |  |
| **Urban** | 13371 | 265.6 | 7095 | | 138.0 | 20466 | 201.2 | | 823 | 16.3 | 246 | 4.79 | 1069 | 10.5 |
| **Town** | 3312 | 247.1 | 1737 | | 124.4 | 5049 | 184.5 | | 213 | 15.9 | 50 | 3.58 | 263 | 9.61 |
| **Village** | 1862 | 141.9 | 1048 | | 79.2 | 2910 | 110.4 | | 180 | 13.7 | 50 | 3.78 | 230 | 8.73 |
| **Quintiles of Welsh Index of Multiple Deprivation** | | | | | | | | |  |  |  |  |  |  |
| **Lowest** | 1992 | 130.0 | 1170 | | 73.8 | 3162 | 101.5 | | 184 | 12.0 | 53 | 3.04 | 237 | 7.60 |
| **Low** | 2689 | 173.3 | 1481 | | 93.2 | 4170 | 132.8 | | 207 | 13.3 | 60 | 3.78 | 267 | 8.50 |
| **Mid** | 3338 | 214.3 | 1864 | | 116.3 | 5202 | 164.6 | | 255 | 16.4 | 68 | 4.24 | 323 | 10.2 |
| **High** | 4358 | 284.4 | 2305 | | 148.3 | 6663 | 215.9 | | 251 | 16.4 | 85 | 5.47 | 336 | 10.9 |
| **Highest** | 6168 | 407.9 | 3060 | | 199.9 | 9228 | 303.3 | | 319 | 21.1 | 80 | 5.23 | 399 | 13.1 |
| **Total** | 18545 | 241.3 | 9880 | | 125.7 | 28425 | 182.8 | | 1216 | 15.8 | 346 | 4.40 | 1562 | 10.0 |
