# Supplementary material for: Risk of suicide following an alcohol-related emergency hospital admission: An electronic cohort study of 2.8 million people
Source: PLoS One. 2018 Apr 27;13(4):e0194772. doi: 10.1371/journal.pone.0194772 (PMC5922531; doi:10.1371/journal.pone.0194772)
Supplement: S2 Table — (DOCX) [file pone.0194772.s002.docx]

**S2 Table: Adjusted risks of death from suicide, stratified by sex**

|  | | **Males** | | | | | | | | | **Female** | | | | **Total** | | | | |
| --- | --- | --- | --- | --- | --- | --- | --- | --- | --- | --- | --- | --- | --- | --- | --- | --- | --- | --- | --- |
|  | | Adjusted HR | | 95.0% CI for HR | | | | | p-value | | Adjusted HR | 95.0% CI for HR | | p-value | Adjusted HR | 95.0% CI for HR | | | p-value |
|  |  |  |  | Lower | | Upper | | |  | |  | Lower | Upper |  |  | Lower | Upper | |  |
| **Sex** | |  | |  | |  | | |  | |  |  |  |  |  |  |  | |  |
| **Female** | |  | |  | |  | | |  | |  |  |  |  | Reference |  |  | |  |
| **Male** | |  | |  | |  | | |  | |  |  |  |  | 3.71 | 3.27 | 4.21 | | <0.001 |
| **Age group** | | | | | | | | | | | | | | | | | | | |
| **10-14** | | 0.19 | | 0.11 | | 0.33 | | <0.001 | | | 0.28 | 0.11 | 0.7 | <0.01 | 0.21 | 0.13 | | 0.33 | <0.001 |
| **16-24** | | Reference | | | | | | | | | Reference | | | | Reference | | | | |
| **15-34** | | 1.47 | | 1.19 | | 1.8 | | <0.001 | | | 1.35 | 0.87 | 2.09 | 0.18 | 1.45 | 1.20 | | 1.74 | <0.001 |
| **35-44** | | 1.83 | | 1.52 | | 2.22 | | <0.001 | | | 1.52 | 1.01 | 2.29 | <0.05 | 1.77 | 1.49 | | 2.11 | <0.001 |
| **45-54** | | 1.37 | | 1.11 | | 1.69 | | <0.01 | | | 2.04 | 1.36 | 3.04 | <0.001 | 1.50 | 1.24 | | 1.8 | <0.001 |
| **55-64** | | 1.04 | | 0.83 | | 1.30 | | 0.76 | | | 1.66 | 1.09 | 2.54 | <0.05 | 1.15 | 0.95 | | 1.41 | 0.16 |
| **65-74** | | 0.84 | | 0.64 | | 1.11 | | 0.22 | | | 1.55 | 0.97 | 2.47 | 0.06 | 0.98 | 0.78 | | 1.24 | 0.87 |
| **75-84** | | 1.40 | | 1.05 | | 1.88 | | 0.02 | | | 2.03 | 1.25 | 3.29 | <0.01 | 1.54 | 1.21 | | 1.98 | <0.001 |
| **85 and over** | | 1.52 | | 0.84 | | 2.73 | | 0.16 | | | 1.25 | 0.53 | 2.98 | 0.61 | 1.37 | 0.85 | | 2.23 | 0.20 |
| **Residential settlement** | | | | | | | | | | | | | | | | | | | |
| **Urban** | Reference | | | | | | | | | Reference | | | | | Reference | | | | |
| **Town** | 1.01 | | 0.87 | | 1.18 | | 0.87 | | | 0.76 | | 0.56 | 1.03 | 0.07 | 0.95 | 0.83 | | 1.09 | 0.49 |
| **Village** | 0.97 | | 0.82 | | 1.16 | | 0.75 | | | 0.86 | | 0.62 | 1.19 | 0.38 | 0.95 | 0.81 | | 1.1 | 0.47 |
| **Quintiles of Welsh Index of Multiple Deprivation** | | | | | | | | | | | | | | | | | | | |
| **Lowest** |  | |  | |  | |  | | | Reference | | | | | Reference | | | | |
| **Low** | 1.11 | | 0.9 | | 1.35 | | 0.33 | | | 1.15 | | 0.79 | 1.67 | 0.46 | 1.12 | 0.93 | | 1.33 | 0.22 |
| **Middle** | 1.34 | | 1.11 | | 1.62 | | <0.01 | | | 1.27 | | 0.88 | 1.82 | 0.20 | 1.32 | 1.12 | | 1.57 | 0.001 |
| **High** | 1.30 | | 1.07 | | 1.57 | | <0.01 | | | 1.57 | | 1.12 | 2.22 | <0.01 | 1.36 | 1.15 | | 1.61 | <0.001 |
| **Highest** | 1.63 | | 1.35 | | 1.95 | | <0.001 | | | 1.44 | | 1.01 | 2.04 | <0.05 | 1.59 | 1.35 | | 1.87 | <0.001 |
| **Alcohol-related admission** | | | | | | | | | | | | | | | | | | | |
| **No** | Reference | | | | | | | | | Reference | | | | | Reference | | | | |
| **Yes** | 9.83 | | 7.91 | | 12.2 | | <0.001 | | | 28.5 | | 19.9 | 40.9 | <0.001 | 26.8 | 18.8 | | 38.3 | <0.001 |
| **Sex * Alcohol-related**  **admission**  **interaction** |  | |  | |  | |  | | |  | |  |  |  | 0.37 | 0.25 | | 0.56 | <0.001 |
